# Metabolic reprogramming‐associated genes predict overall survival for rectal cancer

**DOI:** 10.1111/jcmm.15254

**Published:** 2020-04-13

**Authors:** Zhong‐Yi Zhang, Qing‐Zhi Yao, Hui‐Yong Liu, Qiao‐Nan Guo, Peng‐Jun Qiu, Jian‐Peng Chen, Jian‐Qing Lin

**Affiliations:** ^1^ Departments of Oncological Surgery the Second Affiliated Hospital of Fujian Medical University Quanzhou China

**Keywords:** metabolic reprogramming, prognostic, rectal cancer, survival analysis

## Abstract

Metabolic reprogramming has become a hot topic recently in the regulation of tumour biology. Although hundreds of altered metabolic genes have been reported to be associated with tumour development and progression, the important prognostic role of these metabolic genes remains unknown. We downloaded messenger RNA expression profiles and clinicopathological data from The Cancer Genome Atlas and the Gene Expression Omnibus database to uncover the prognostic role of these metabolic genes. Univariate Cox regression analysis and lasso Cox regression model were utilized in this study to screen prognostic associated metabolic genes. Patients with high‐risk demonstrated significantly poorer survival outcomes than patients with low‐risk in the TCGA database. Also, patients with high‐risk still showed significantly poorer survival outcomes than patients with low‐risk in the GEO database. What is more, gene set enrichment analyses were performed in this study to uncover significantly enriched GO terms and pathways in order to help identify potential underlying mechanisms. Our study identified some survival‐related metabolic genes for rectal cancer prognosis prediction. These genes might play essential roles in the regulation of metabolic microenvironment and in providing significant potential biomarkers in metabolic treatment.

## 1 INTRODUCTION

Surgeons have been focused on colorectal cancer for a long time, and lots of scientific researches have been focused on the development and progression of colorectal cancer. Nevertheless, there are some differences in aetiology between colon cancer and rectal cancer.^1^ Bailey et al^2^ demonstrated that the age‐related incidence rates of rectal cancer are dramatically increased in the United States over the past 25 years. Also, it has been estimated that by 2030, 22.9% of all rectal cancers will be diagnosed in patients under the screening age compared to 9.5% in 2010. Plus, the incidence rates of rectal cancer increased from 6.51 and 7.68 to 8.28 and 11.45 per 100 000 in females and males in China, respectively.^1^ Therefore, it is meaningful to put more concentration on molecular changes to uncover the biological process of rectal cancer.^3^

Metabolic reprogramming has increasingly become a hot topic recently. Catabolic and anabolic metabolism is essential for cancer cells to ensure their energy supply and biomass synthesis by reprogramming their microenvironments.^4^, ^5^, ^6^, ^7^ Previous studies^8^, ^9^, ^10^ have been discussed the emerging importance role of dysregulated metabolism for cancer biology. However, the underlying processes and molecular alterations of metabolic programming in cancers have not been well elucidated yet, especially in rectal cancer.

In this study, we thought to focus on the important metabolic alterations in rectal cancer through analysing data downloaded from the TCGA database and validated the conclusions by the GEO database. The significant prognostic role of metabolic genes in rectal cancer has been conducted in this study.

## 2 METHODS

### 2.1 mRNA expression profiles and clinical information

We downloaded mRNA expression profiles and corresponding clinical information from The Cancer Genome Atlas (TCGA) database, which contains a total of 91 cases. We then downloaded 363 cases from the GEO database and obtained the gene expression matrix of the GSE87211 series.^11^ The metabolic genes were obtained from the GSEA website (http://software.broadinstitute.org/gsea/index.jsp), downloads section. GSEA v4.0.3 for Windows and c2.cp.kegg.v7.0.symbols.gmt were downloaded from the GSEA website for the further extraction analyses.

### 2.2 Extraction of metabolic genes from the TCGA database and GEO database

Genes enriched in metabolism pathways of the KEGG database were utilized in this study as metabolic genes. The mRNA expression of metabolic genes in the TCGA database and GEO database was extracted, respectively. Then, an adjustment was given to adjust different mRNA expression levels of metabolic genes between TCGA and GEO databases by ‘sva’ package^12^, ^13^ in R software (version 3.6.1). Genes were selected as candidate metabolic genes for the following analysis if: (a) they have the same expression pattern in TCGA database and the GEO database; and (b) they were listed in the metabolic‐associated pathways.

### 2.3 Identification of differentially expressed metabolic genes

Candidate metabolic genes have the same expression pattern both in the TCGA and GEO databases were selected. We utilized the R package ‘limma’ to screen the differentially expressed metabolic genes.^14^ The expression of candidate metabolic genes in the TCGA database was used to identify differentially expressed metabolic genes. The screening criteria were set as |logFC| > 0.5 and *P*‐value < .05.

### 2.4 Identification of prognostic associated metabolic genes

Univariate Cox regression analysis was utilized in this study to identify prognostic associated metabolic genes in the TCGA database. Genes with HR < 1 have better overall survival outcomes, while genes with HR > 1 have worse overall survival outcomes. Genes with *P* < .05 were regarded as prognostic associated metabolic genes.

### 2.5 Construction of lasso Cox regression model

Prognostic associated metabolic genes screened by univariate Cox regression analysis were utilized to construct the lasso Cox regression model.^15^ Lasso Cox regression model was constructed aimed to calculate the risk score of every patient. The formula of risk score was as follows: riskscore = the sum of each coefficient of mRNA multiple each expression of mRNA. Patients were divided into two groups based on the median risk score of each patient.

### 2.6 Survival analysis based on the stratification of low‐ and high‐risk scores in TCGA database

Patients were divided into two groups based on the median risk score. Survival analysis was performed by the Kaplan‐Meier method. Risk score curves were generated based on the risk score of each patient.

### 2.7 Validation of TCGA survival analysis by utilizing data from the GEO database

In order to validate the survival data in the TCGA database, we downloaded survival data from the GEO database to perform Kaplan‐Meier analysis.

### 2.8 Validation of risk score in the TCGA database by univariate and multivariate Cox analysis

The clinicopathological characteristics and risk score were included in univariate and multivariate Cox regression analysis to validate whether the risk score can be regarded as an independent risk factor to predict overall survival outcome. ROC curve was used to assess the constructed model.

### 2.9 GO and KEGG analyses by GSEA

We utilized GESA software^16^ (version 4.0.3, permutations was set as 1000) to perform GO and KEGG analyses. ftp.broadinstitute.org://pub/gsea/gene_sets/c2.cp.kegg.v7.0.symbols.gmt and ftp.broadinstitute.org://pub/gsea/gene_sets/c5.all.v7.0.symbols.gmt were selected as gene sets databases, respectively.

## 3 RESULTS

### 3.1 Extraction of metabolic genes from the TCGA database

We downloaded a total of 91 cases from the TCGA database. A total of 804 metabolic genes were extracted from the TCGA gene expression matrix, including 375 up‐regulated metabolic genes and 429 down‐regulated metabolic genes. We then downloaded 363 cases from the GEO database and obtained the gene expression matrix of the s series.

### 3.2 Identification of differentially expressed metabolic genes

**Figure 1:**
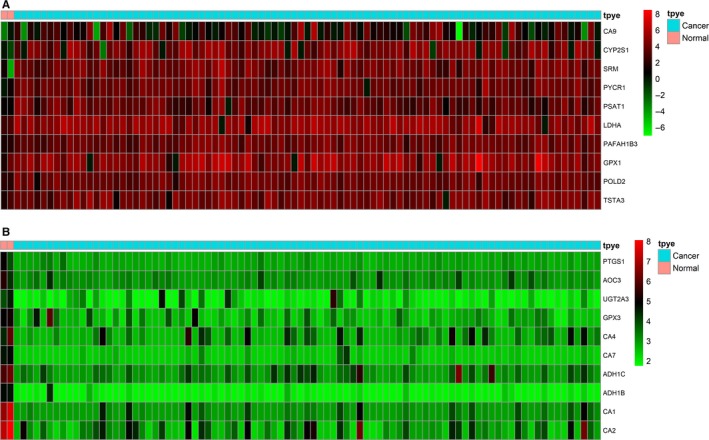
The heatmaps of differentially expressed genes. A, The heatmap of the top 10 up‐regulated genes. B, The heatmap of the top 10 down‐regulated genes

After screening, we identified 154 differentially expressed metabolic genes in the TCGA database under the screening criteria (|logFC| > 0.5 and the *P*‐value < .05), including 65 up‐regulated metabolic genes and 89 down‐regulated metabolic genes. The top 10 differentially up‐regulated metabolic genes and the top 10 differentially down‐regulated metabolic genes were demonstrated in Table 1. The heatmaps of these top differentially up‐regulated and down‐regulated metabolic genes were demonstrated in Figure 1.

**Table 1:** The top 10 up‐regulated and top 10 down‐regulated genes

Gene	ConMean	TreatMean	LogFC	*P*‐value
Up‐regulated genes				
CA9	0.05829499	8.192816882	7.134843859	.033573543
CYP2S1	0.19855646	25.30546893	6.993756099	.040879028
SRM	1.25746506	21.03853542	4.064444057	.016589274
PYCR1	1.18208029	17.66674515	3.901636332	.017854753
PSAT1	1.935826165	14.78036715	2.932660796	.022172087
LDHA	6.66345163	39.9240846	2.582917745	.031392164
PAFAH1B3	3.133727225	17.60374774	2.489931092	.016589274
GPX1	9.032856135	47.70557092	2.400903613	.049687304
POLD2	3.962352605	17.59980148	2.151129981	.022172087
TSTA3	5.428126315	20.66517161	1.928675146	.038373038
Down‐regulated genes				
PTGS1	23.26466006	7.223703527	−1.687329518	.016589274
AOC3	30.80712967	9.283017238	−1.730598569	.019204026
UGT2A3	19.3533035	5.643672461	−1.77787368	.02553134
GPX3	32.28367394	8.139981238	−1.987707395	.020641585
CA4	44.02872034	10.31134722	−2.094212075	.035912432
CA7	27.33950795	5.604131221	−2.286424636	.016589274
ADH1C	66.74952942	11.75626673	−2.505327669	.019204026
ADH1B	27.03287165	4.085192341	−2.726238864	.016584958
CA1	151.8035669	9.806519947	−3.952320623	.016588842
CA2	209.5200422	12.62820428	−4.052366847	.016589274

### 3.3 Identification of prognostic associated metabolic genes

**Figure 2:**
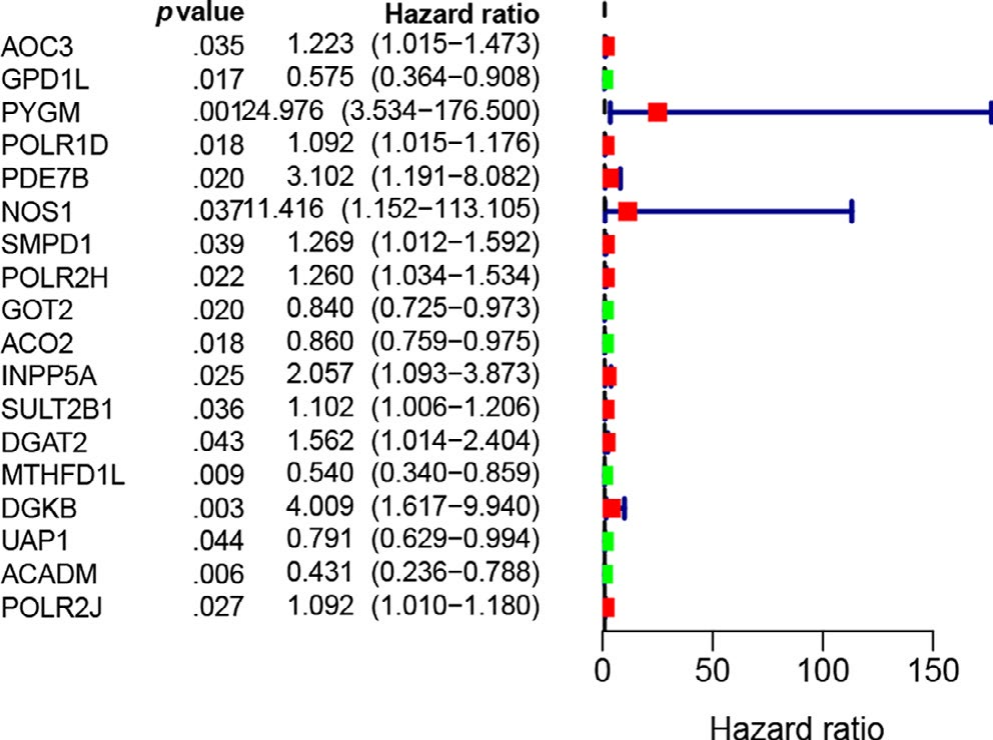
The forest plot of the prognostic related metabolic genes screened by univariate Cox regression analysis

A total of 18 prognostic associated metabolic genes were identified, including PYGM, NOS1, DGKB, PDE7B, INPP5A, DGAT2, SMPD1, POLR2H, AOC3, SULT2B1, POLR1D, POLR2J, ACO2, GOT2, UAP1, GPD1L, MTHFD1L and ACADM. Among them, PYGM, NOS1, DGKB, PDE7B, INPP5A, DGAT2, SMPD1, POLR2H, AOC3, SULT2B1, POLR1D and POLR2J were associated with higher risks of poor overall survival outcomes, while ACO2, GOT2, UAP1, GPD1L, MTHFD1L and ACADM were associated with lower risks of poor overall survival outcomes (Figure 2).

### 3.4 Construction of lasso Cox regression model

A total of 11 prognostic associated metabolic genes were included in the lasso Cox regression model, including PYGM, DGAT2, INPP5A, POLR1D, POLR2H, DGKB, SULT2B1, SMPD1, GOT2, MTHFD1L and ACADM. The riskscore = 2.0420*expression of PYGM + 0.2823*expression of DGAT2 + 0.1504*expression of INPP5A + 0.0954*expression of POLR1D + 0.0876*expression of POLR2H + 0.0854*expression of DGKB + 0.0291*expression of SULT2B1 + 0.0208*expression of SMPD1‐0.0449*expression of GOT2‐0.2101*expression of MTHFD1L‐0.3224*expression of ACADM.

### 3.5 Validation of TCGA survival analysis by utilizing data from the GEO database

**Figure 3:**
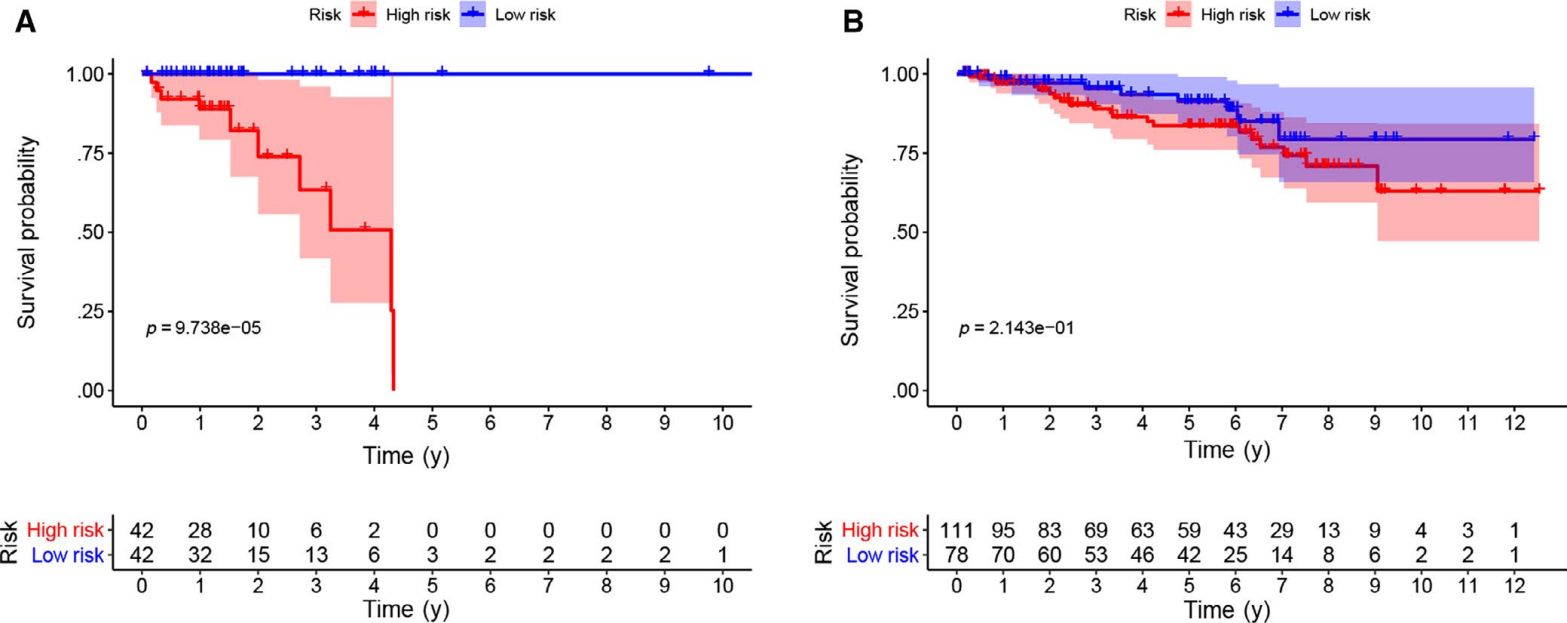
Survival analyses for the prognostic metabolic genes in rectal cancer. A, The Kaplan‐Meier curve showed the survival of patients was significantly poorer in patients with high‐risk in the TCGA database. B, The Kaplan‐Meier curve showed the survival of patients was significantly poorer in patients with high‐risk in the GEO database

Kaplan‐Meier analysis demonstrated that high‐risk group had a worse overall survival outcome compared with the low‐risk group (Figure 3A). Also, data from the GEO database showed that high‐risk group had a worse overall survival outcome (Figure 3B).

### 3.6 Validation of risk score, survival status distribution and heatmap of the TCGA database by utilizing data from the GEO database

**Figure 4:**
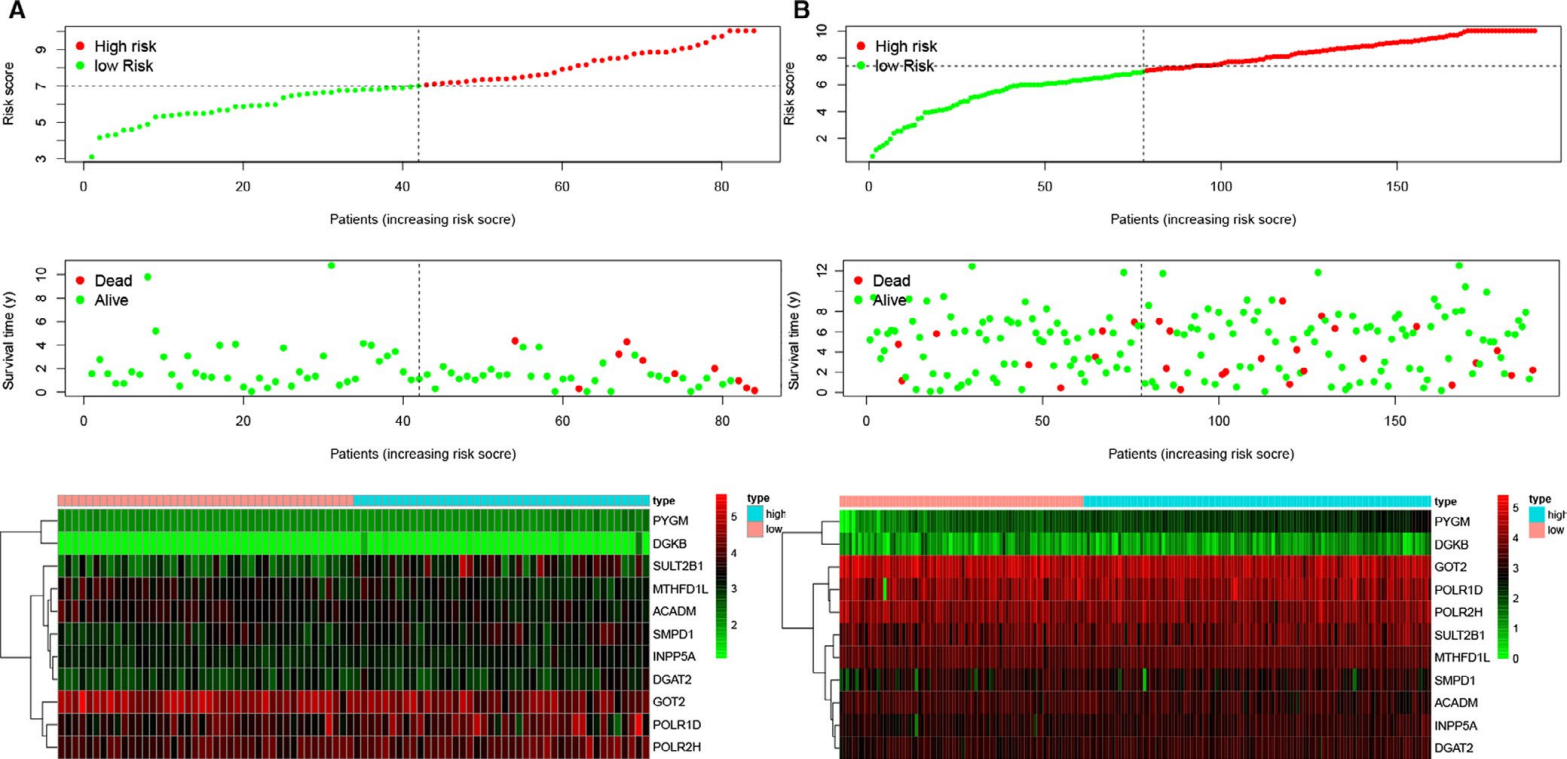
The risk score analysis, survival status distribution analysis and heatmap for metabolic genes in rectal cancer. A, The risk score analysis, survival status distribution analysis and heatmap in the TCGA database. B, The risk score analysis, survival status distribution analysis and heatmap in the GEO database

On top of Figure 4A,B, every patient was ranked from left to right according to the risk score. The risk score was elevated from left to right in both TCGA and the GEO database. Also, in the middle of Figure 4A,B, every patient was ranked from left to right according to the risk score. The distribution of the survival status of each patient has demonstrated accordingly. Plus, at the bottom of Figure 4A,B, the heatmaps demonstrated the differential expression of metabolic genes in high‐risk and low‐risk groups in the TCGA database (Figure 4A) and GEO database (Figure 4B).

### 3.7 Univariate and multivariate Cox analysis in the TCGA database

**Figure 5:**
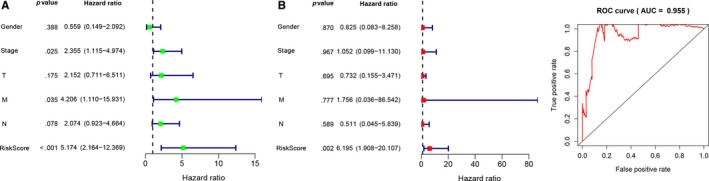
Forrest plot of the univariate and multivariate Cox regression analysis and evaluation of receiver operating curve (ROC) analysis in rectal cancer by TCGA database. A, Forrest plot of the univariate Cox regression analysis. B, Forrest plot of the multivariate Cox regression analysis. C, The efficacy of the multivariate Cox regression analysis was evaluated by ROC analysis

Univariate Cox regression analysis demonstrated that the risk score was associated with the overall survival of rectal cancer patients. With the increasing risk score, the risk of poor survival outcomes elevated (Figure 5A). The results of the multivariate Cox regression analysis showed that the risk score could be treated as an independent risk factor to predict overall survival outcome in rectal cancer patients (Figure 5B). The results of the ROC curve demonstrated that the constructed predictive model has robust predictive value in predicting overall survival (Figure 5C).

### 3.8 GO and KEGG analyses by GSEA

**Figure 6:**
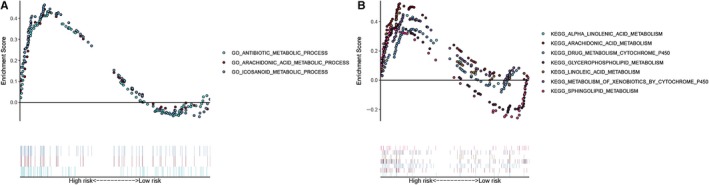
The GO terms and KEGG pathways enriched by GSEA were demonstrated. A, Three representative metabolic‐associated GO terms in high‐risk patients. B, Seven representative metabolic‐associated KEGG pathways in high‐risk patients

The results of GO analysis demonstrated that genes were mainly enriched in antibiotic metabolic process, arachidonic acid metabolic process and icosanoid metabolic process (Figure 6A). The results of KEGG analysis showed that alpha‐linolenic acid metabolism, arachidonic acid metabolism, drug metabolism cytochrome p450, glycerophospholipid metabolism, linoleic acid metabolism, metabolism of xenobiotics by cytochrome p450 and sphingolipid metabolism pathways (Figure 6B).

## 4 DISCUSSION

The standard treatment for rectal cancer remains the total mesorectal excision.^17^ Nevertheless, patients are requesting gradually for better‐tailored managements for rectal cancer due to the developments of neoadjuvant therapy and multidisciplinary team management.^18^, ^19^, ^20^ Since rectal cancer surgery often accompanied by high morbidity and poor oncologic outcomes, surgeons are gradually focusing on the molecular alterations in rectal cancer in order to find some valid targets in the treatment of rectal cancer.^21^, ^22^, ^23^

Recently, metabolic reprogramming has become a hot topic.^24^ Accumulating evidence demonstrates the influence of metabolic alterations in neoplastic cells, mainly focused on the different cellular components of the cell microenvironment, like the regulation of tumour‐infiltrating immune cells.^25^, ^26^, ^27^ Therefore, the identification of novel metabolic genes in cancer to predict mortality risk of cancer has become a hotspot. In this study, we screened novel metabolic prognostic related genes from the TCGA database and validated the efficiency by data downloaded from the GEO database. The low‐risk and high‐risk patients were effectively stratified based on the different overall survival outcomes. The efficacy was further validated by data from the GEO database, which indicates a robust prognostic value of the prediction efficacy of these metabolic‐related genes.

GSEA was utilized in this study to uncover the enriched GO terms and pathways in rectal cancer. Most GO terms and pathways were metabolism‐related. On the one hand, the results enriched in GO terms and pathways proved the robust connection of the screened metabolic‐related genes and indicated the underlying dysregulated metabolic microenvironment in rectal cancer. Patients with high‐risk were more likely to be associated with the metabolism GO terms, like antibiotic metabolic process, arachidonic acid metabolic process and icosanoid metabolic process. Plus, patients with high‐risk were more likely to be connected with the metabolism pathways, including alpha‐linolenic acid metabolism, arachidonic acid metabolism, drug metabolism cytochrome p450, glycerophospholipid metabolism, linoleic acid metabolism, metabolism of xenobiotics by cytochrome p450 and sphingolipid metabolism pathways. Therefore, we hypothesized that high‐risk patients might benefit more from metabolic therapy and metabolic‐related management. Also, these results uncovered the underlying molecular mechanisms of these prognostic metabolic genes. However, many works are needed to further validate the relationship between these genes and metabolic treatment and related managements. Here, Peng et al^8^ showed that the expression of the metabolic genes is correlated with the mutations and copy number changes that occur in many cancers include those of the rectum. Sinkala et al^9^ showed that colorectal cancer cell lines with alterations to genes of various metabolic pathways tend to be more responsive to multiple anticancer drugs than those without alterations to the metabolic genes. Competently to these findings, Peng et al^8^ further showed that the metabolic expression subtypes of cancers are informative about drug sensitivities.

A total of 11 prognostic associated metabolic genes were included in the lasso Cox regression model, including PYGM, DGAT2, INPP5A, POLR1D, POLR2H, DGKB, SULT2B1, SMPD1, GOT2, MTHFD1L and ACADM. The risk score formula was as follow: 2.0420*expression of PYGM + 0.2823*expression of DGAT2 + 0.1504*expression of INPP5A + 0.0954*expression of POLR1D + 0.0876*expression of POLR2H + 0.0854*expression of DGKB + 0.0291*expression of SULT2B1 + 0.0208*expression of SMPD1‐0.0449*expression of GOT2‐0.2101*expression of MTHFD1L‐0.3224*expression of ACADM. We have classified these 11 genes into three groups. The first group is the reported colorectal cancer‐associated metabolic genes group. The second group is a cancer‐associated metabolic gene group. The third group is the unreported gene group. The genes in the reported colorectal cancer group include POLR1D, DGKB, SULT2B1 SMPD1 GOT2, MTHFD1L and ACADM. The genes in the second group include DGAT2, INPP5A and POLR2H. Nevertheless, only one metabolic gene, PYGM, among these 11 genes has not been reported to be associated with the development and progress of cancer.

POLR1D has been reported to be associated with the promotion of colorectal cancer progression and prediction of poor prognosis of patients.^28^ Zhou et al^29^ demonstrated that the DGKB gene region was only opened in SW620‐AA cells. Li et al^30^ showed that SULT2B1 was significantly elevated in colorectal cancer tissues than in normal tissues. Also, they found that SULT2B1 played a crucial role in the oestrogen metabolic pathway and to be associated with colorectal cancer risk and survival. SMPD1 could be treated as the critical prognostic biomarker in colorectal cancer.^31^ GOT2 was reported by Du et al^32^ that ectopic expression of GOT2 could attenuate the SOX12 knock‐down‐induced suppression of colorectal cancer progression. Plus, MTHFD1L was reported by Agarwal et al^33^ that involved in the progression of colorectal cancer. Also, ACADM might be involved in the regulation of the overall survival of colorectal cancer.

DGAT2 has been reported to be associated with hepatocellular carcinoma malignancy by regulation of the cell cycle‐related gene expression.^34^ Also, DGAT2 has been reported to be associated with the regulation of the development of prostate cancer.^35^ INPP5A could be treated as the target of miR‐181a‐5p in regulation on the progression of cervical cancer.^36^ POLR2H was reported to be associated with the progression of prostate cancer.^37^

Our results have identified some prognostic related metabolic genes for predicting survival outcomes of rectal cancer based on TCGA data. Some results could be validated by data downloaded from the GEO database, which reflected that these genes might be involved in the dysregulation of the metabolic microenvironment and might be treated as novel biomarkers for metabolic therapy.

## CONFLICT OF INTEREST

The authors declared that they have no conflicts of interest to this work.

## AUTHOR CONTRIBUTIONS

ZYZ and JQL involved in study concept and design; QZY, HYL, QNG and PJQ involved in acquisition of data; QZY, HYL, QNG, PJQ and JPC involved in analysis and interpretation of data. ZYZ, QZY and JQL drafted the manuscript; ZYZ, QZY and JQL involved in critical revision of the manuscript for intellectual content.

## Data Availability

The data that support the findings of this study are openly available in The Cancer Genome Atlas and the Gene Expression Omnibus (GSE87211) at http://www.tcga.org/ and https://www.ncbi.nlm.nih.gov/geo/, respectively.
